# The Editor's Letter-Box

**Published:** 1920-07-31

**Authors:** 


*To the Editor of* The Hospital.

Sir,—For your spacious hospitality many thanks. The editorial comment on my letter is absolutely fair and reasoned. The problem *is* complex.

May I more exactly put my point of view thus: Does it seem workable—scientific—to disregard the very obvious fact that every system and function has its specific and indispensable office, or to distort our machine from its idiom to fit into the contemporary mode of life? Will this effect progressive evolution?—Yours, etc.

"Contemplator."

